# OTUD7B exacerbates atherosclerosis by promoting RIPK1-dependent vascular smooth muscle cell necroptosis

**DOI:** 10.3389/fcvm.2026.1749659

**Published:** 2026-06-18

**Authors:** Linfei Yu, Yulong Wang, Yiheng Li, Yu Zhang

**Affiliations:** 1 Department of Cardiology, Haiyan People’s Hospital, Jiaxing, China; 2 Department of Cardiology, The Second Affiliated Hospital Zhejiang University School of Medicine, Hangzhou, China

**Keywords:** atherosclerosis, inflammation, necroptosis, OTUD7B, RIPK1-RIPK3-MLKL axis

## Abstract

**Background:**

Atherosclerosis is a vascular disease characterized by lipid deposition, chronic inflammation, and cell death. Necroptosis, a form of programmed necrosis, plays a critical role in atherosclerosis progression. This study investigates the expression of the deubiquitinating enzyme OTUD7B in atherosclerosis and its mechanism in regulating vascular injury via the RIPK1-mediated necroptosis pathway.

**Methods:**

An atherosclerosis model was established in mice fed a high-fat diet combined with partial carotid ligation. OTUD7B and necroptosis-related proteins RIPK1, RIPK3, and phosphorylated MLKL (p-MLKL) were detected in arterial tissues. *In vitro*, a necroptosis model was induced in human aortic smooth muscle cells (HA-SMCs) using the TSZ protocol. OTUD7B was knocked down to assess cell viability, inflammatory cytokine levels, and necroptosis. OTUD7B knockdown and RIPK1 co-overexpression were employed to validate its protective effects and dependency *in vivo*.

**Results:**

OTUD7B was significantly upregulated in plaques of atherosclerosis mice, concomitant with increased expression and phosphorylation of RIPK1 and RIPK3, as well as elevated p-MLKL levels. TUNEL staining and ELISA confirmed elevated necroptosis and inflammation. *In vitro*, OTUD7B knockdown markedly alleviated HA-SMC injury, suppressed the expression and phosphorylation of necroptosis markers, and reduced IL-1β/TNF-α release. *In vivo* OTUD7B knockdown attenuated plaque formation, lipid deposition, necroptosis, and inflammation. Mechanistically, RIPK1 overexpression significantly reversed the protective effects of OTUD7B knockdown, restoring RIPK1/RIPK3 phosphorylation and downstream signaling, indicating its functional dependency on the RIPK1 pathway. Co-immunoprecipitation further confirmed a direct OTUD7B-RIPK1 interaction, facilitating downstream signaling activation.

**Conclusion:**

OTUD7B exacerbates necroptosis and inflammation in vascular smooth muscle cells by activating the RIPK1-RIPK3-MLKL axis, thereby playing a detrimental role in AS. This study identifies OTUD7B as a potential therapeutic target for atherosclerosis intervention.

## Introduction

Atherosclerosis (AS), the primary pathological basis of cardiovascular diseases, is characterized by aberrant lipid deposition within the arterial wall, chronic inflammation, and vascular cell dysfunction (1). These processes culminate in life-threatening clinical events such as myocardial infarction and stroke, significantly contributing to global morbidity and mortality (2). While established risk factors include hypercholesterolemia, hypertension, diabetes, and smoking, atherosclerosis is fundamentally recognized as a chronic vascular inflammatory condition triggered by the interplay between these factors and arterial wall cells (3). Vascular endothelial cells, vascular smooth muscle cells (VSMCs), and macrophages play pivotal roles in the development of atherosclerotic plaques and subsequent atherosclerosis progression (4). Despite current clinical interventions, such as statins and antiplatelet therapies, which can slow atherosclerosis progression, significant unmet needs persist. These include high plaque vulnerability and post-interventional restenosis (5), underscoring the urgent requirement for novel therapeutic targets.

Dysregulation of cell death modalities represents a critical driver of atherosclerotic plaque formation and rupture (6). Necroptosis, a caspase-independent programmed form of cell necrosis, occurs within advanced atherosclerotic lesions and has been implicated in the dysfunction of VSMCs (7). Emerging evidence indicates that necroptosis, mediated by a signaling cascade involving receptor-interacting protein kinase 1/3 (RIPK1/RIPK3) and mixed lineage kinase domain-like protein (MLKL), exacerbates the vascular inflammatory microenvironment and tissue damage (8). This occurs through plasma membrane rupture and the release of pro-inflammatory cytokines such as interleukin-1β (IL-1β) and tumor necrosis factor-α (TNF-α) (9). In atherosclerosis models, targeted inhibition of the necroptosis pathway effectively reduces plaque necrotic core area and enhances plaque stability, highlighting its potential as a therapeutic intervention point (10). However, the intricate regulatory mechanisms governing this pathway in AS, particularly the influence of post-translational modifications like ubiquitination on its activity, remain incompletely elucidated.

The dynamic equilibrium of ubiquitination and deubiquitination is a central mechanism regulating protein stability and function, extensively involved in pathophysiological processes including cell death and inflammation (11). The deubiquitinating enzyme (DUB) OTUD7B (ovarian tumor domain-containing protein 7B), also known as Cezanne, is a cell cycle-regulated DUB. While OTUD7B has been demonstrated to regulate key signaling pathways through substrate deubiquitination in cancers and immune disorders (12, 13), its role in cardiovascular diseases remains unexplored. Notably, the ubiquitination status of RIPK1 directly determines the activation threshold for necroptosis. K63-linked polyubiquitination promotes RIPK1 activation, whereas K48-linked ubiquitination targets it for proteasomal degradation (14). Based on this evidence, we hypothesize that OTUD7B may accelerate atherosclerosis progression by positively regulating the necroptosis pathway through the specific deubiquitination of RIPK1.

This study aims to elucidate the biological function of OTUD7B in atherosclerosis and its underlying molecular mechanism. By integrating apolipoprotein E-deficient (*Apoe^−^/^−^*) mouse models of atherosclerosis with VSMC necroptosis models, and employing conditional gene knockdown, overexpression, and protein interaction validation experiments, we systematically investigate the regulatory role of OTUD7B on the RIPK1/RIPK3/MLKL signaling axis. Our findings will not only provide novel insights into the necroptosis regulatory network within atherosclerosis but also establish a theoretical foundation for developing targeted therapeutic strategies focused on the OTUD7B-RIPK1 axis.

## Methods

### Animal housing

Six-week-old male C57BL/6JNifdc-Apoe^em1Vr/Vr mice (hereafter referred to atherosclerosis *Apoe^−^/^−^* mice) were obtained from Beijing Vital River Laboratory Animal Technology Co., Ltd. The mice were housed under specific pathogen-free (SPF) conditions in a controlled environment maintained at 22–25°C with 60%–65% relative humidity and a standardized 12-hour light/dark cycle. Prior to experimental procedures, all animals underwent a one-week acclimation period within this facility.

### Animal grouping and modeling

A total of 36 *Apoe^−^/^−^* mice were randomly assigned to six experimental groups (*n* = 6): Control, AS, atherosclerosis + sh-NC, atherosclerosis + sh-OTUD7B, atherosclerosis + sh-OTUD7B + oe-NC, and atherosclerosis + sh-OTUD7B + oe-RIPK1. All animals received a high-fat diet [HFD, D12079B, SPF (Beijing) Biotechnology Co., Ltd., China; 41% kcal from fat, 0.15% cholesterol] for six weeks prior to modeling to establish a baseline atherosclerosis condition.

The surgical procedure followed established methodologies (15). Under anesthesia induced by inhalation of 2% isoflurane (803250, McKesson, Texas, USA), mice underwent tandem stenosis of the right common carotid artery (CCA). A midline cervical incision was made, followed by dissection of the connective tissue surrounding the right CCA. Titanium wires (150 µm diameter) were threaded around the vessel 1 mm distal to the carotid bifurcation and 3 mm proximal to the distal stenosis site. External ligation was performed using 6–0 blue braided polyester sutures to secure each wire, after which the wires were removed.

To achieve VSMC-specific knockdown of OTUD7B or overexpression of RIPK1, custom-engineered adeno-associated virus serotype 9 (AAV9) vectors (supplied by Shanghai GeneChem Co., Ltd.) were utilized. These vectors contained expression cassettes driven by the SM22α promoter (AAV9-SM22α-shOTUD7B or AAV9-SM22α-RIPK1). Viral suspension (50 µL, titer: 1 × 10^11 ^v.g./mL) was administered via tail vein injection.

Postoperatively, animals were maintained on the HFD for an additional six weeks. At the experimental endpoint, euthanasia was performed via intraperitoneal injection of an overdose of pentobarbital sodium (200 mg/kg; P3761, Sigma-Aldrich, St. Louis, Missouri, USA). Tissue specimens were subsequently harvested for downstream analyses.

#### Serum lipid analysis

Blood samples were collected from mice via cardiac puncture at the time of euthanasia. Serum was separated by centrifugation at 3,000 rpm for 15 min at 4°C and stored at −80°C until analysis. Serum levels of total cholesterol (TC), triglycerides (TG), and low-density lipoprotein cholesterol (LDL-C) were measured using an automatic biochemical analyzer (Hitachi 7,100, Japan) according to the manufacturer's instructions.

### Hematoxylin and eosin (HE) staining

Right CCAs harvested from mice were fixed in 4% paraformaldehyde solution (158,127, Sigma-Aldrich) for 24 h. Following fixation, tissues underwent sequential dehydration, clearing, and paraffin embedding. Sections were deparaffinized in xylene (422685000, Thermo Fisher Scientific, Waltham, MA, USA) for 5 min and rehydrated through a graded ethanol series (445740010, Thermo Fisher Scientific). Tissue sections were subsequently stained with hematoxylin (HHS16, Sigma-Aldrich) for 5 min, differentiated in 1% acid-alcohol solution, and blued in 0.2% ammonium hydroxide for 1 min. Counterstaining was performed using eosin solution (HT110132, Sigma-Aldrich) for 1 min. Stained sections were then dehydrated through ethanol, cleared in xylene, and permanently mounted with neutral resin mounting medium (HX93203, Thermo Fisher Scientific). Histological evaluation was conducted using a Nikon Eclipse E200 microscope (Nikon Corporation, Tokyo, Japan).

### Oil red O staining

Right CCA tissues from mice were fixed in 4% paraformaldehyde for 24 h, dehydrated, and embedded in optimal cutting temperature (OCT) compound (6478.2, Carl Roth, Karlsruhe, Germany). Serial 5-μm-thick cryosections were prepared at −20°C. A working staining solution was prepared by mixing saturated oil red O solution (HY-D1168, MedChemExpress, New Jersey, USA) with distilled water in a 3:2 (v/v) ratio followed by filtration. Cryosections were stained with the working solution at room temperature for 6 h, then sequentially rinsed three times with 60% isopropanol (383910025, Thermo Fisher Scientific) and once with running water. Nuclei were counterstained with hematoxylin for 2 min followed by a final water rinse. Sections were permanently mounted and imaged using a Leica microscope system (Leica Microsystems, Germany). Lipid plaque areas were quantified using ImageJ software (National Institutes of Health, Bethesda, MD, USA).

### Reverse transcription-quantitative polymerase chain reaction (RT-qPCR) for OTUD7B detection

Total RNA was isolated using TRIzol reagent (15596026, Thermo Fisher Scientific). RNA concentration was quantified spectrophotometrically using an HD-UV90 instrument (Shandong Hold Electronic Technology Co., Weifang, China). For cDNA synthesis, 2 μg of total RNA underwent RT with the SynScript® III One-Step RT Kit (DLR102, Vazyme Biotech Co., Ltd., Nanjing, China). Real-time qPCR amplification was performed on a thermal cycler (Applied Biosystems, Foster City, CA, USA). Relative gene expression levels were calculated using the 2^–ΔΔCt^ method, with glyceraldehyde-3-phosphate dehydrogenase (GAPDH) serving as the reference gene. Primer sequences are provided in Table 1.

**Table 1:** The primer sequences for RT-qPCR.

Gene	Primer sequences (5′–3′)	
Mmu-OTUD7B	Forward	GGAGGTGAAGTTACATCTGCTGC
	Reverse	TCAGGAGTGGACCTGGGTTCAT
Mmu-GAPDH	Forward	CATCACTGCCACCCAGAAGACTG
	Reverse	ATGCCAGTGAGCTTCCCGTTCAG
Hsa-OTUD7B	Forward	TCTCAGAGGCTGCTTCCTTTGG
	Reverse	CGCCTTTTCAACGCTTCCTTCTC
Hsa-GAPDH	Forward	GTCTCCTCTGACTTCAACAGCG
	Reverse	ACCACCCTGTTGCTGTAGCCAA

### Western blot (WB) analysis

Cells were lysed in RIPA buffer (R0278, Sigma-Aldrich) for 30 min on ice with gentle agitation every 5 min. Lysates were centrifuged at 12,000 rpm for 10 min at 4°C, and supernatants were collected. Protein concentrations were determined using a BCA protein assay kit (23,227, Thermo Fisher Scientific). Proteins were separated by sodium dodecyl sulfate-polyacrylamide gel electrophoresis (SDS-PAGE) and subsequently transferred to polyvinylidene difluoride (PVDF) membranes (88,518, Thermo Fisher Scientific). Membranes were blocked with 5% non-fat milk for 1 h at room temperature, followed by overnight incubation at 4°C with the following primary antibodies: anti-OTUD7B (1:1,000, SAB2101695, Sigma-Aldrich), anti-RIPK1 (1:1,000, PA5-20811, Thermo Fisher Scientific), anti-RIPK3 (1:1,000, PA5-141114, Thermo Fisher Scientific), anti-phospho-RIPK1 (Ser166) (1:1,000, 28252-1-AP, Proteintech), anti-phospho-RIPK3 (Ser232) (1:1,000, 87148-1-RR, Proteintech; for mouse tissues), anti-phospho-RIPK3 (Ser227) (1:1,000, 13555, Signalway Antibody; for human cells), anti-phosphorylated MLKL (p-MLKL, 1:1,000, PA5-105678, Thermo Fisher Scientific), and anti-GAPDH (1:1,000, G9545, Sigma-Aldrich) serving as the loading control. After washing, membranes were probed with horseradish peroxidase (HRP)-conjugated anti-rabbit secondary antibody (1:20,000, 31,464, Thermo Fisher Scientific) for 1 h at room temperature. Protein bands were detected using enhanced chemiluminescence (ECL) reagent (32,106, Thermo Fisher Scientific), and band intensity was quantified with ImageJ software.

### Immunohistochemical staining

Paraffin-embedded tissue sections underwent antigen retrieval in sodium citrate buffer (C9999, Sigma-Aldrich). Non-specific binding sites were blocked with 5% bovine serum albumin (BSA) blocking solution (A9647, Sigma-Aldrich) for 30–60 min at room temperature. Sections were subsequently incubated overnight at 4°C with primary antibody against p-MLKL (PA5-105678, Thermo Fisher Scientific). The next day, sections were probed with HRP-conjugated secondary antibody (1:1,000, 31,470, Thermo Fisher Scientific) for 30 min. Color development was achieved using 3,3′-diaminobenzidine (DAB) chromogenic substrate (SK-4100, Vector Laboratories, Burlingame, CA, USA). Nuclei were counterstained with hematoxylin, followed by dehydration and permanent mounting. Stained sections were examined microscopically, with quantitative image analysis performed using ImageJ software.

### TUNEL staining for necroptosis detection

Paraffin-embedded sections of murine right CCAs were processed for TUNEL staining to assess necroptotic cell death. Following sequential deparaffinization and rehydration, sections were treated with 20 μg/mL proteinase K (HY-108717, MedChemExpress) for 20 min at 37°C to enhance tissue permeability. Enzymatic labeling was then performed according to manufacturer's specifications using the TUNEL assay kit (C10618, Invitrogen, Carlsbad, CA, USA). Nuclei were counterstained with 4′,6-diamidino-2-phenylindole (DAPI; 62,248, Thermo Fisher Scientific) for 10 min at room temperature under light-protected conditions. After final phosphate-buffered saline (PBS) washes, sections were imaged under a fluorescence microscope at 200× magnification. For quantitative analysis, five non-overlapping fields per slide (each containing ≥ 100 cells) were randomly selected. The necroptosis index was calculated as follows: Necroptosis rate = (Number of TUNEL-positive nuclei/Total nuclei counted) × 100%.

### Enzyme-linked immunosorbent assay (ELISA)

Serum levels of IL-1β (88-7013A-88, Invitrogen, Waltham, MA, USA) and TNF-α (BMS607-3, Invitrogen) in mice, along with IL-1β (BMS224-2, Invitroge) and TNF-α (88-7346-88, Invitroge) concentrations in cell culture supernatants, were quantified using commercial ELISA kits according to the manufacturer's protocols.

### Cell culture and necroptosis modeling

Human aortic smooth muscle cells (HA-SMCs) were maintained in high-glucose Dulbecco's modified Eagle medium (DMEM; 11965092, Gibco™, Waltham, MA, USA) supplemented with 10% fetal bovine serum (A5670701, Gibco™), 100 U/mL penicillin, and 100 μg/mL streptomycin (15070063, Gibco™). Cells were incubated at 37°C in a humidified 5% CO₂ atmosphere. To induce necroptosis, cells were stimulated using the TSZ protocol as previously described (16, 17). Briefly, following 30-minute pretreatment with Z-VAD-FMK (20 μM; HY-16658B, MedChemExpress) and SM-433 (10 nM; HY-138059, MedChemExpress), cells were challenged with human recombinant TNF-α (20 ng/mL, SRP3177, Sigma-Aldrich) for 15 h to activate the necroptotic pathway.

For genetic manipulation experiments, subconfluent cells (70%–80% confluence) were transduced with lentiviral vectors carrying specific constructs (VectorBuilder, Chicago, IL, USA) at a multiplicity of infection (MOI) of 10 for 48 h. The constructs included: short hairpin negative control (sh-NC), OTUD7B-targeting shRNA (sh-OTUD7B), overexpression empty vector (oe-NC), and RIPK1 overexpression vector (oe-RIPK1). Lentiviral particles had titers of approximately 1 × 10^8^ transducing units (TU)/mL. Transduction efficiency was validated by qPCR 48 h post-infection. Stable cell lines were subsequently selected through 48-hour treatment with puromycin (2 μg/mL).

### Cell viability assessment via cell counting kit-8 (CCK-8) assay

Cellular viability was evaluated using the CCK-8 (C0037, Beyotime Biotechnology, Shanghai, China). Cells were seeded into 96-well plates at a density of 3 × 10^3^ cells per well. Following PBS washes, 10 μL of CCK-8 reagent was combined with 90 μL of serum-free medium per well. The plates were subsequently incubated for 2 h at 37°C in a humidified atmosphere containing 5% CO_2_. Absorbance was measured at 450 nm using a microplate reader (BioTek Instruments Inc., Winooski, VT, USA) to quantify viable cells.

### Flow cytometric analysis of necroptosis

Cell necroptosis was evaluated using the ApoDETECT Annexin V-FITC/PI dual-staining kit (331,200, Thermo Fisher Scientific) according to manufacturer specifications. Briefly, harvested cells were trypsinized and neutralized with complete medium, followed by centrifugation at 1,000 rpm for 5 min. After supernatant aspiration, cells were resuspended at 1 × 10^6 ^cells/mL in binding buffer. Aliquots of 100 µL cell suspension were incubated with 5 µL Annexin V-FITC and 5 µL propidium iodide (PI) working solution for 10 min under light-protected conditions at room temperature. Stained cells were immediately subjected to analysis on a CytoFLEX LX flow cytometer (Beckman Coulter, Inc., Brea, CA, USA).

### Co-immunoprecipitation (Co-IP) assay

Cells were harvested and lysed in ice-cold lysis buffer supplemented with protease inhibitors, followed by incubation on ice for 30 min. Lysates were subjected to high-speed centrifugation to remove cellular debris, and supernatants were collected. The supernatants were incubated overnight at 4°C with anti-OTUD7B antibody (SAB2101695, Sigma-Aldrich) or rabbit immunoglobulin G (IgG) isotype control (31,235, Invitrogen). Protein A/G magnetic beads (88,802, Thermo Fisher Scientific) were then added to the antigen-antibody complexes and incubated for 2 h. Following three washes with ice-cold lysis buffer, the bead-bound complexes were resuspended in sample buffer and denatured by boiling for 5 min. Eluted proteins were subsequently analyzed by WB.

### Statistical analysis

All statistical analyses were performed using Prism 9 software (GraphPad Software, San Diego, CA, USA). Continuous data were presented as mean ± standard deviation. Intergroup comparisons were evaluated by unpaired *t*-test for two groups, while one-way or two-way analysis of variance followed by Tukey's *post hoc* analysis was applied for multiple group comparisons. Statistical significance was defined as *P* < 0.05.

## Results

### OTUD7B expression is upregulated in atherosclerosis lesions with concomitant enhancement of necroptosis signaling

**Figure 1:**
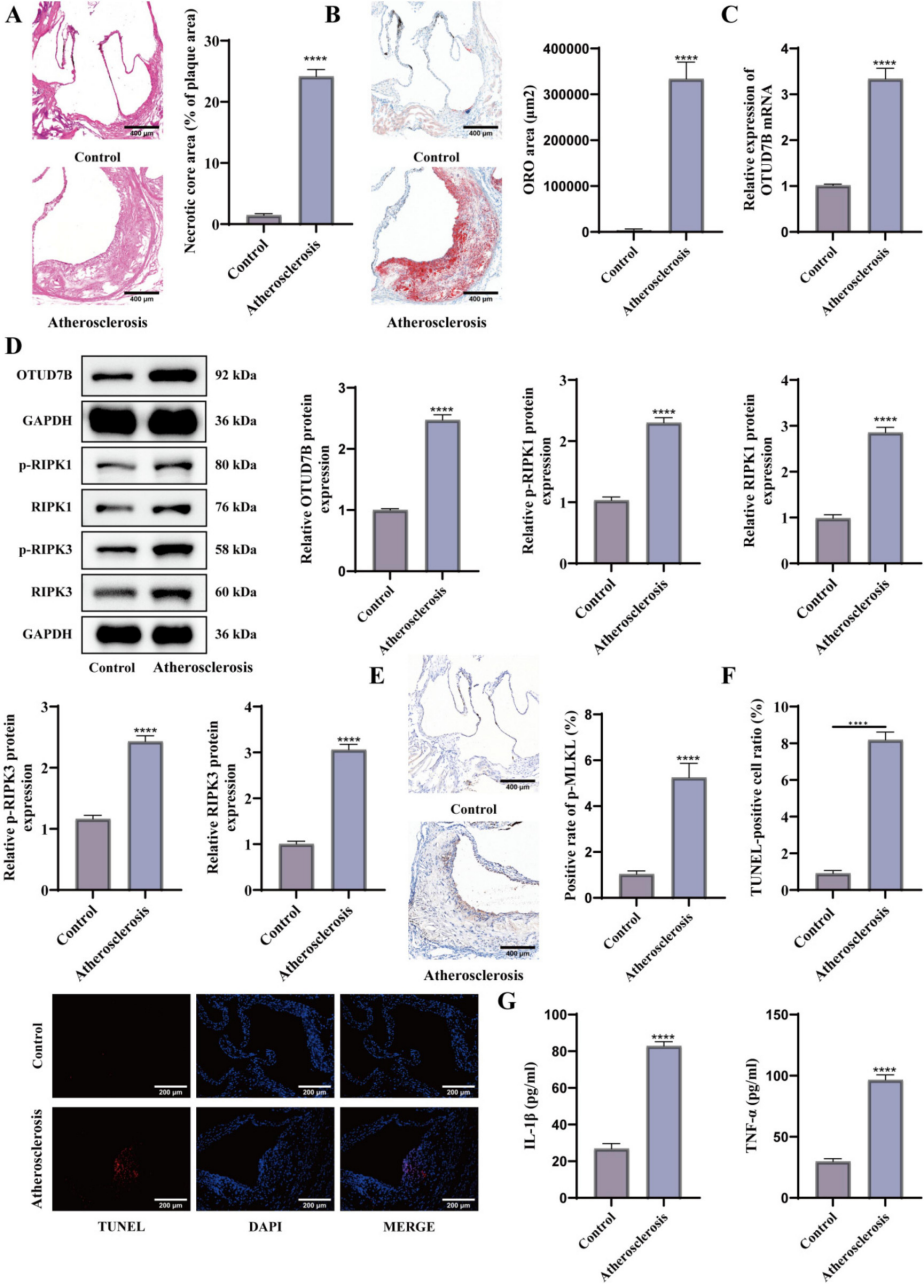
OTUD7B upregulation in atherosclerotic tissues is associated with enhanced necroptosis signaling. **(A)** HE staining of pathological alterations in carotid arteries and quantitative analysis. **(B)** Lipid deposition assessed by oil red O staining and quantitative analysis. **(C)** OTUD7B mRNA expression detected by RT-qPCR. **(D)** Protein levels and phosphorylation of OTUD7B, p-RIPK1, RIPK1, p-RIPK3, and RIPK3 evaluated by WB. **(E)** Immunohistochemical detection of p-MLKL expression. **(F)** Vascular wall cell death measured via TUNEL staining. **(G)** Serum IL-1β and TNF-α concentrations quantified by ELISA. (*n* = 6). ****P < 0.0001. A t-test was used for comparisons between two groups.

To investigate the expression dynamics and potential role of OTUD7B in AS, an experimental model was established using *Apoe^−^/^−^* mice subjected to HFD feeding and CCA tandem stenosis. HE staining revealed substantial atherosclerotic plaque formation in the arterial walls of the atherosclerosis group, characterized by disorganized medial architecture and extensive foam cell infiltration, with quantitative analysis further demonstrating a significantly larger necrotic core area compared to the Control group (Figure 1A). Oil red O staining further demonstrated significantly augmented lipid deposition within lesioned areas (Figure 1B). RT-qPCR analysis indicated marked upregulation of OTUD7B mRNA expression in the atherosclerosis model **(**Figure 1C), while WB confirmed increased OTUD7B protein levels alongside significantly elevated expression and phosphorylation of necroptosis-associated mediators RIPK1 and RIPK3 (Figure 1D). Immunohistochemical analysis revealed enhanced p-MLKL expression predominantly localized around necrotic cores and within medial regions of plaques in the atherosclerosis group (Figure 1E), indicative of necroptosis activation. This finding was corroborated by TUNEL staining, which showed substantially increased vascular wall necroptosis in the atherosclerosis group (Figure 1F). ELISA additionally detected significantly elevated serum levels of IL-1β and TNF-α in atherosclerosis mice compared with controls (Figure 1G). To exclude the possibility that systemic lipid metabolism contributes to the observed atherosclerotic changes, we measured serum lipid levels in Control and atherosclerosis groups. atherosclerosis shown in Supplementary Figure S1, no significant differences were observed in serum TC, TG, or LDL-C levels between the two groups, indicating that all *Apoe^−^/^−^* mice fed a high-fat diet exhibited comparable hyperlipidemic backgrounds. These results suggest that the differences in plaque formation are attributable to local vascular regulation rather than systemic lipid metabolism. Collectively, these results demonstrate that atherosclerosis lesions exhibit significant OTUD7B upregulation concomitant with activation of RIPK1/RIPK3/p-MLKL-mediated necroptosis and inflammatory responses, suggesting OTUD7B may accelerate atherosclerosis progression by potentiating necroptotic cell death and inflammation.

### OTUD7B knockdown attenuates necroptosis in VSMCs

**Figure 2:**
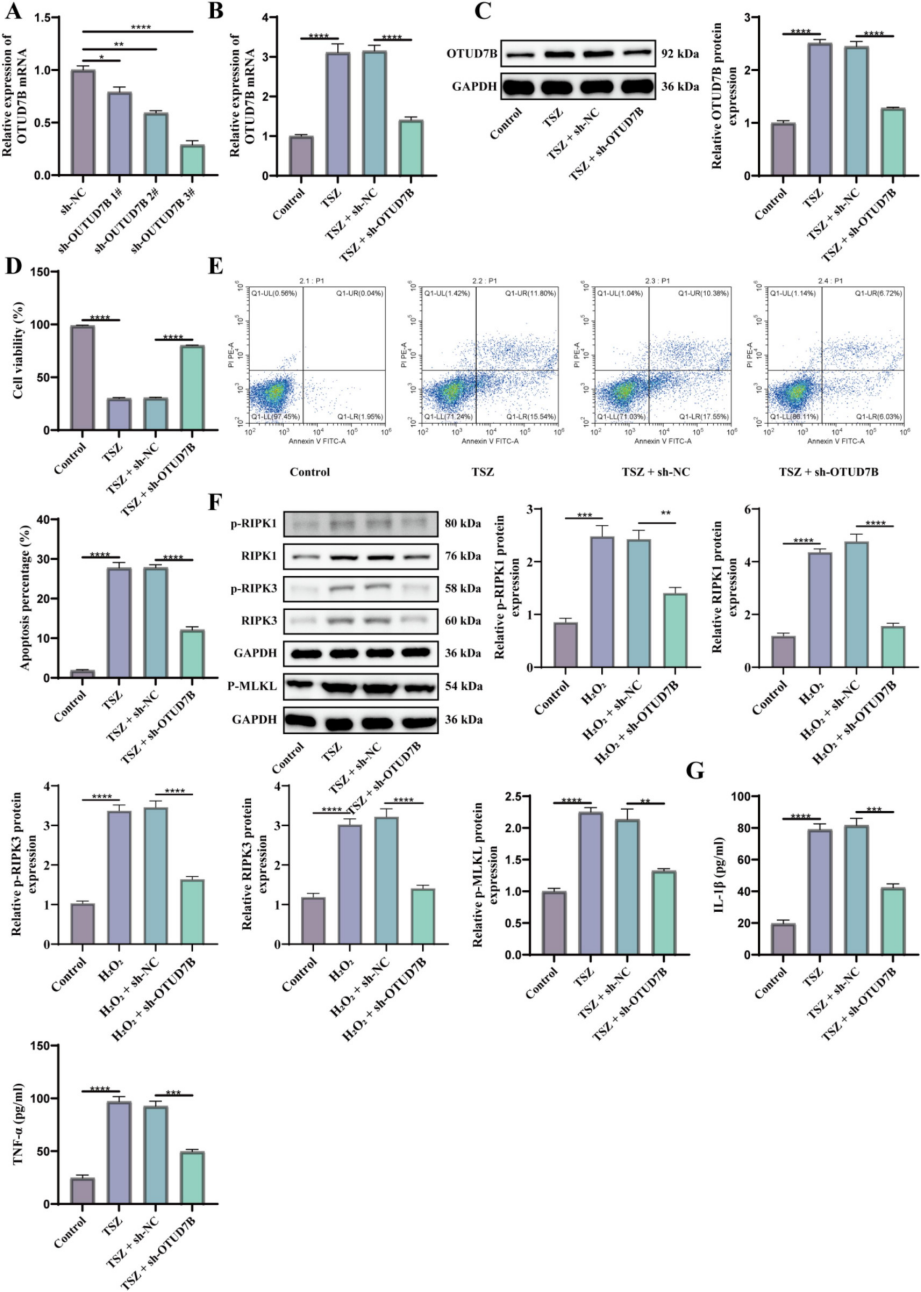
OTUD7B knockdown inhibits necroptosis in VSMCs. Human aortic smooth muscle cells (HA-SMCs) were treated with TSZ after lentiviral transduction with sh-NC or sh-OTUD7B. **(A)** shRNA screening efficiency assessed by RT-qPCR. **(B,C)** OTUD7B knockdown efficiency validated by RT-qPCR and WB. **(D)** Cellular viability measured via CCK-8 assay. **(E)** Proportion of necroptotic cells quantified by flow cytometry. **(F)** Protein levels and phosphorylation of p-RIPK1, RIPK1, p-RIPK3, RIPK3, and p-MLKL analyzed by WB. **(G)** IL-1β and TNF-α concentrations in cell supernatants determined by ELISA. (*n* = 3). *P < 0.05, **P < 0.01, ***P < 0.001, ****P < 0.0001. For three or more groups, one-way analysis of variance (ANOVA) was applied, followed by Tukey's *post hoc* test.

To delineate the functional role of OTUD7B in VSMC necroptosis, a necroptosis model was established in HA-SMCs using the TSZ (TNF-α + SM-433 + Z-VAD-FMK) induction protocol, followed by lentivirus-mediated OTUD7B knockdown. Screening of shRNA efficiency via RT-qPCR identified sh-OTUD7B 3# as the optimal construct, which was subsequently employed for all functional experiments (Figure 2A). Both RT-qPCR and WB analyses confirmed significant TSZ-induced upregulation of OTUD7B expression, which was effectively suppressed by sh-OTUD7B transduction (Figures 2B,C). Functional assessment using CCK-8 assay revealed that TSZ stimulation substantially compromised cellular viability, whereas OTUD7B knockdown partially ameliorated this TSZ-induced cytotoxicity (Figure 2D). Flow cytometric analysis demonstrated a marked reduction in the proportion of necroptotic cells following OTUD7B silencing under TSZ challenge (Figure 2E). At the molecular level, WB analysis demonstrated that TSZ treatment upregulated the expression and phosphorylation of key necroptotic mediators RIPK1 and RIPK3, as well as p-MLKL levels, effects that were significantly attenuated by OTUD7B depletion (Figure 2F). Furthermore, ELISA detected reduced secretion of proinflammatory cytokines IL-1β and TNF-α in the supernatant of OTUD7B-deficient cells after TSZ stimulation (Figure 2G). Collectively, these findings indicate that OTUD7B potentiates TSZ-induced necroptosis and inflammatory responses in VSMCs, suggesting its pathogenic contribution to vascular pathophysiology.

### OTUD7B knockdown attenuates atherosclerosis progression and suppresses necroptosis *in vivo*

**Figure 3:**
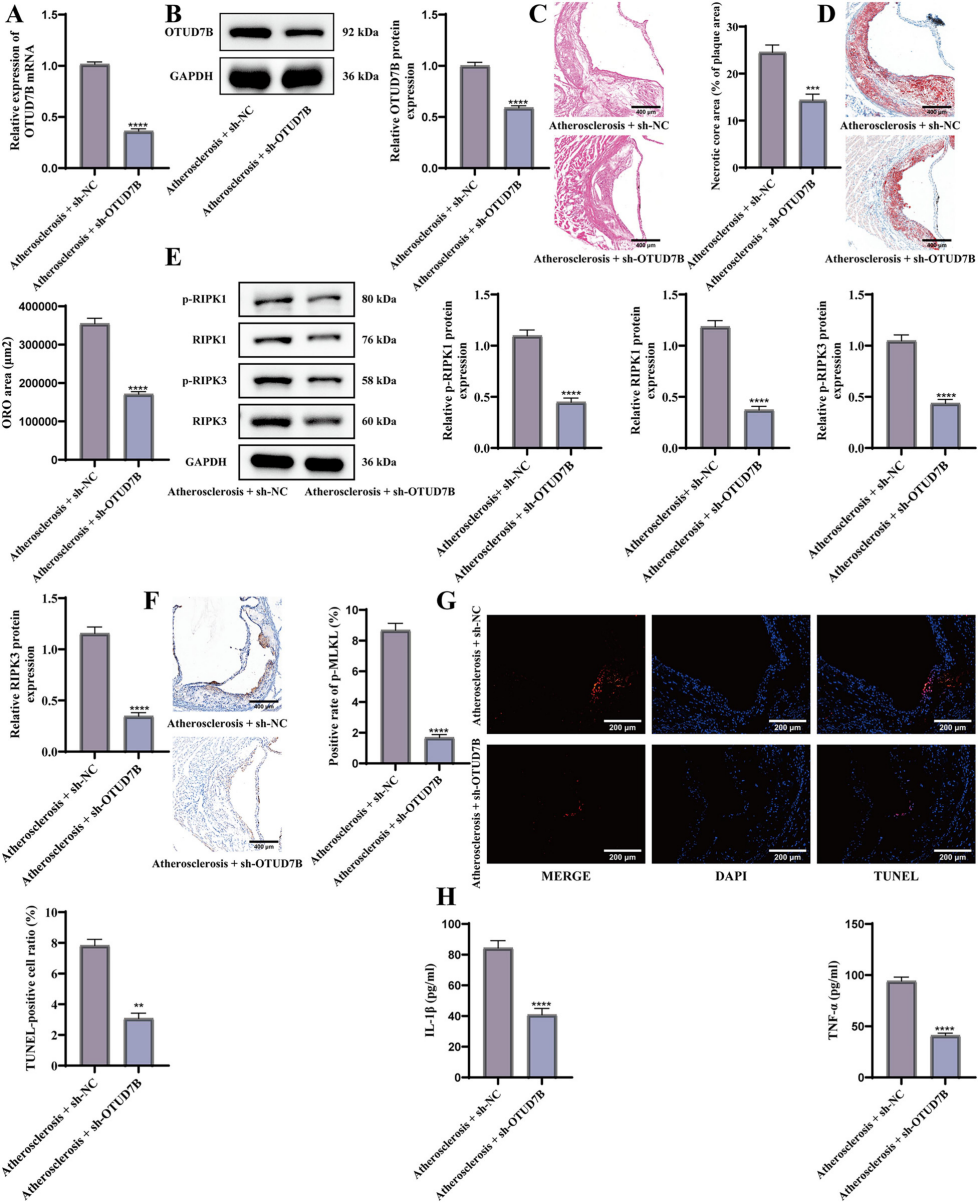
OTUD7B knockdown attenuates atherosclerosis progression and necroptosis *in vivo*. **(A,B)** OTUD7B knockdown efficiency in vascular tissues assessed by RT-qPCR and WB. **(C)** HE staining of carotid arteries and quantification of necrotic core area. **(D)** Lipid deposition quantified via oil red O staining. **(E)** Protein levels and phosphorylation of p-RIPK1, RIPK1, p-RIPK3, and RIPK3 analyzed by WB. **(F)** p-MLKL expression detected by immunohistochemistry. **(G)** Proportion of necroptotic cells measured via TUNEL staining. **(H)** Serum IL-1β and TNF-α concentrations determined by ELISA. (*n* = 6). ***P < 0.001, ****P < 0.0001. A t-test was used for comparisons between two groups.

To validate the regulatory role of OTUD7B in atherosclerosis pathogenesis, *Apoe^−^/^−^* mice received tail vein injections of AAV9-SM22α-shOTUD7B for VSMC-specific knockdown. RT-qPCR and WB analyses confirmed efficient OTUD7B depletion in vascular tissues (Figures 3A,B). HE staining revealed consolidated plaque architecture with attenuated medial disruption in the atherosclerosis + sh-OTUD7B group, with quantitative analysis further demonstrating a significantly reduced necrotic core area compared to the atherosclerosis + sh-NC group (Figure 3C). Oil red O staining demonstrated substantially reduced lipid deposition in this intervention group, indicating amelioration of atherosclerotic pathology (Figure 3D). WB analysis showed that OTUD7B knockdown significantly reduced both the expression and phosphorylation of key necroptotic mediators RIPK1 and RIPK3 (Figure 3E). Immunohistochemical analysis further confirmed diminished expression of p-MLKL within lesional areas (Figure 3F). TUNEL staining revealed decreased proportions of necroptotic cells in the vascular wall (Figure 3G). ELISA additionally detected reduced serum concentrations of proinflammatory cytokines IL-1β and TNF-α (Figure 3H). Collectively, these *in vivo* findings demonstrate that OTUD7B exacerbates atherosclerosis progression, principally through potentiation of RIPK1/RIPK3/MLKL-mediated necroptosis and associated inflammation.

### OTUD7B modulates necroptosis through the RIPK1-mediated signaling axis

**Figure 4:**
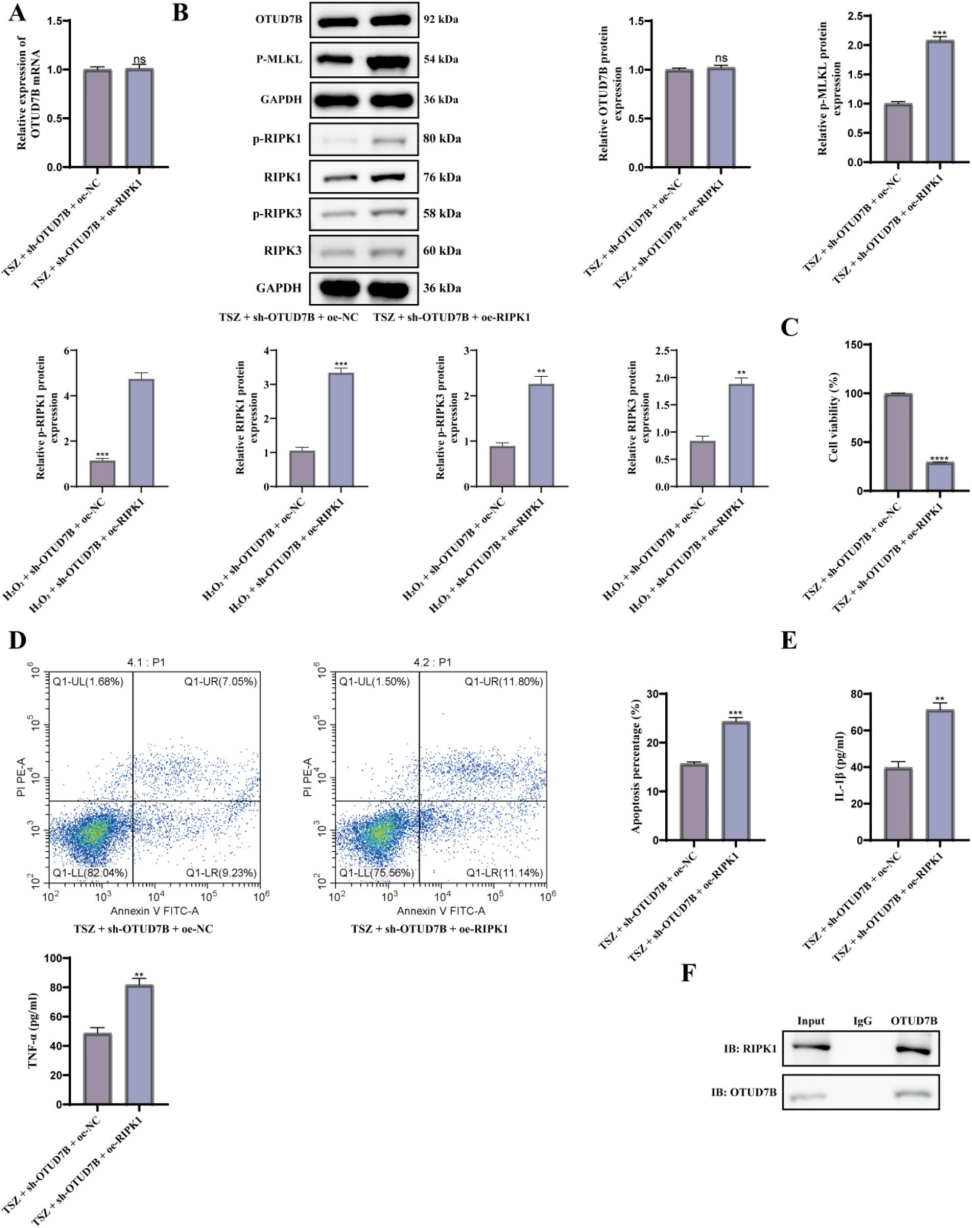
OTUD7B regulates necroptosis via the RIPK1-mediated pathway. HA-SMCs were transduced with the indicated lentiviruses and then stimulated with TSZ. **(A)** OTUD7B mRNA expression detected by RT-qPCR. **(B)** Protein levels and phosphorylation of OTUD7B, p-RIPK1, RIPK1, p-RIPK3, RIPK3, and p-MLKL analyzed by WB. **(C)** Cellular viability assessed via CCK-8 assay. **(D)** Necroptosis-specific cell death quantified by flow cytometry. **(E)** IL-1β and TNF-α concentrations measured by ELISA. **(F)** Protein interaction between OTUD7B and RIPK1 detected by Co-IP. (*n* = 3). ns *P* > 0.05, **P < 0.01, ***P < 0.001, ****P < 0.0001. A t-test was used for comparisons between two groups.

To elucidate the precise mechanism underlying OTUD7B-regulated necroptosis, HA-SMCs were subjected to TSZ-induced necroptosis modeling coupled with RIPK1 overexpression. RT-qPCR analysis confirmed the efficient knockdown of OTUD7B at the transcriptional level in the designated experimental groups (Figure 4A). WB analysis demonstrated that RIPK1 upregulation in OTUD7B-knockdown cells significantly restored the phosphorylation of RIPK1 and RIPK3, as well as the downstream p-MLKL expression, without affecting OTUD7B knockdown efficiency (Figure 4B), establishing RIPK1 as the pivotal upstream regulator of this pathway. Functional assessment via CCK-8 assay revealed that RIPK1 overexpression substantially abrogated the protective effect of OTUD7B silencing on cellular viability (Figure 4C). Flow cytometric quantification further confirmed that RIPK1 reconstitution reversed OTUD7B knockdown-mediated suppression of necroptosis (Figure 4D), collectively indicating RIPK1's central role in OTUD7B-dependent cell death regulation. ELISA detected proportional elevation of proinflammatory cytokines IL-1β and TNF-α following RIPK1 restoration (Figure 4E), consistent with necroptosis-associated inflammatory amplification. Crucially, Co-IP assays verified direct protein-protein interaction between OTUD7B and RIPK1 (Figure 4F), indicating that OTUD7B promotes necroptotic signaling through targeted RIPK1 modulation. These findings establish a mechanistic paradigm wherein OTUD7B exacerbates vascular cellular damage by facilitating RIPK1-dependent activation of the RIPK1/RIPK3/MLKL necroptotic cascade and associated inflammatory responses.

### Protective effect of OTUD7B knockdown depends on RIPK1 pathway activity

**Figure 5:**
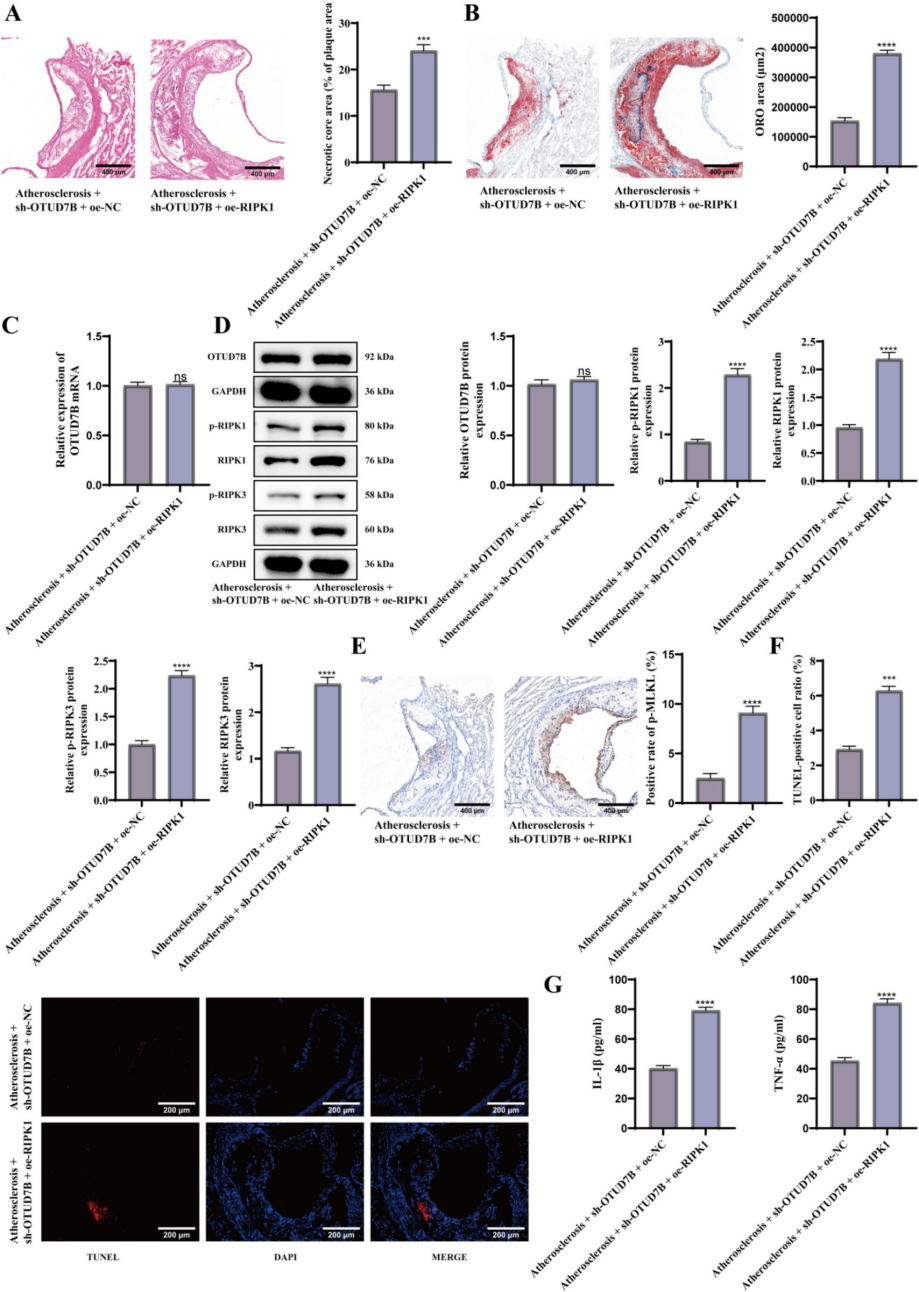
The protective effect of OTUD7B knockdown depends on RIPK1 pathway activity. **(A)** HE staining of carotid arteries and quantification of necrotic core area. **(B)** Lipid deposition quantified via oil red O staining. **(C)** OTUD7B mRNA expression analyzed by RT-qPCR. **(D)** Protein levels and phosphorylation of OTUD7B, p-RIPK1, RIPK1, p-RIPK3, and RIPK3 analyzed by WB. **(E)** p-MLKL expression detected by immunohistochemistry. **(F)** Necroptotic cells in vascular walls measured via TUNEL staining. **(G)** Serum IL-1β and TNF-α concentrations determined by ELISA. (*n* = 6). ns *P* > 0.05, ***P < 0.001, ****P < 0.0001. A *t*-test was used for comparisons between two groups.

To determine whether OTUD7B knockdown confers protection against atherosclerosis through RIPK1 pathway modulation, *Apoe^−^/^−^* mice underwent concurrent RIPK1 overexpression. HE staining revealed prominent atherosclerotic plaque formation with disorganized medial architecture in the RIPK1-overexpressing cohort (AS + sh-OTUD7B + oe-RIPK1) compared to controls (AS + sh-OTUD7B + oe-NC), with quantitative analysis further confirming a significantly increased necrotic core area, indicating partial abrogation of OTUD7B knockdown-mediated vascular remodeling benefits (Figure 5A). Oil red O staining demonstrated increased lipid deposition and significantly expanded plaque areas following RIPK1 upregulation (Figure 5B). RT-qPCR and WB analyses confirmed comparable OTUD7B mRNA and protein expression between groups, whereas RIPK1 and its downstream effector RIPK3 were substantially upregulated at both the total protein and phosphorylation levels in RIPK1-overexpressing mice, validating pathway activation (Figures 5C,D). Immunohistochemical detection revealed enhanced expression of p-MLKL, a necroptosis executioner, predominantly localized in intimal and medial layers of vascular plaques, indicating augmented necroptotic activity (Figure 5E). TUNEL staining confirmed elevated proportions of necroptotic cells in the vascular wall upon RIPK1 restoration (Figure 5F). ELISA additionally detected significantly elevated serum concentrations of proinflammatory cytokines IL-1β and TNF-α (Figure 5G). Collectively, RIPK1 overexpression substantially attenuated the atheroprotective effects of OTUD7B knockdown *in vivo*, demonstrating that OTUD7B downregulation mitigates vascular pathology through suppression of RIPK1-mediated necroptosis and inflammation.

## Discussion

AS arises from complex interplay among dysregulated lipid metabolism, chronic inflammation, and aberrant activation of cell death pathways (18). Necroptosis, a precisely regulated form of programmed necrosis, plays a pivotal role in atherosclerosis progression. It triggers plasma membrane rupture, releasing pro-inflammatory cytokines (e.g., IL-1β, TNF-α), thereby exacerbating the vascular inflammatory microenvironment and promoting plaque instability (19). The present study is the first to uncover the pathogenic function of the deubiquitinating enzyme OTUD7B in AS. We demonstrate that OTUD7B directly binds to and activates RIPK1, driving the RIPK1/RIPK3/MLKL signaling axis. This activation promotes VSMC necroptosis and inflammatory cascades, ultimately accelerating atherosclerosis progression. Our findings not only expand the understanding of the molecular regulatory network underlying atherosclerosis but also identify a novel potential therapeutic target within the necroptosis pathway.

We initially observed significant upregulation of OTUD7B at both mRNA and protein levels in *Apoe^−^/^−^* mouse atherosclerosis models and HA-SMCs subjected to necroptosis induction. Importantly, this elevated OTUD7B expression correlated positively with the activation of necroptosis markers (RIPK1, RIPK3, p-MLKL) and the release of pro-inflammatory cytokines (IL-1β, TNF-α), suggesting its potential involvement in atherosclerosis pathogenesis. While OTUD7B, a novel deubiquitinating enzyme, has been primarily studied in oncogenesis, where it regulates the stability of oncoproteins or tumor suppressors through deubiquitination (13, 20), emerging evidence supports our findings. OTUD7B expression levels are reported to be consistently induced by various atherogenic stimuli in VSMCs and remodeled arteries upon injury. Higher OTUD7B expression is also observed in human VSMCs and atherosclerotic lesions, and it functions as a key regulator of VSMC proliferation and migration during pathological arterial remodeling in atherosclerosis (21). Notably, VSMC dedifferentiation, proliferation, and migration are hallmark features of vasculature dysregulation and significantly contribute to atherosclerotic plaque development (22). The work by An et al. provides compelling evidence that OTUD7B holds potential for limiting VSMC proliferation and associated vascular disease (23). Furthermore, OTUD7B has been shown as a potentially effective therapeutic strategy against fibrosis following myocardial infarction (24).

Our functional loss-of-function experiments, both *in vitro* and *in vivo*, robustly confirmed the pro-atherogenic role of OTUD7B. Specific knockdown of OTUD7B significantly inhibited TSZ-induced necroptosis in HA-SMCs, improved cell viability, and reduced pro-inflammatory cytokine secretion. In *Apoe^−^/^−^* mice, VSMC-specific OTUD7B knockdown effectively attenuated atherosclerotic plaque burden, lipid deposition, and medial structural disruption, concurrently reducing necroptosis levels and systemic inflammation. These results definitively establish OTUD7B as a driver of atherosclerosis pathology and highlight its potential as a therapeutic intervention target.

The core necroptosis signaling pathway depends on RIPK1 activation and its subsequent cascade involving RIPK3 and MLKL (25). Activated RIPK1 recruits RIPK3 via homotypic interactions to form the necrosome complex. This complex then phosphorylates MLKL, leading to its oligomerization, translocation to the plasma membrane, membrane disruption, and ultimately, cell lysis and pro-inflammatory mediator release (26, 27). MLKL acts as the executioner of necroptosis, and its phosphorylation marks the definitive activation of this pathway (28, 29). Our mechanistic investigations revealed that OTUD7B knockdown significantly suppressed both the expression and phosphorylation of RIPK1 and RIPK3, as well as MLKL phosphorylation. Crucially, overexpression of RIPK1 reversed the protective effects conferred by OTUD7B knockdown against both necroptosis and atherosclerosis lesion formation, unequivocally identifying RIPK1 as the core downstream effector of OTUD7B function. Most importantly, Co-IP experiments confirmed a direct physical interaction between OTUD7B and RIPK1, providing a structural basis for OTUD7B-mediated regulation of RIPK1.

Based on OTUD7B's intrinsic deubiquitinating enzyme activity and its direct binding to RIPK1, it is plausible that OTUD7B modulates RIPK1 stability and function by editing its ubiquitination status, although direct evidence for this mechanism is currently lacking. Existing evidence indicates that the ubiquitination state of RIPK1 is critical for determining its engagement in apoptotic vs. necroptotic pathways (30). For instance, M1-linked linear ubiquitination mediated by the linear ubiquitin chain assembly complex suppresses RIPK1's necroptotic activity (31), while the deubiquitinase CYLD promotes RIPK1 activation by removing K63-linked ubiquitin chains (32). Our finding that OTUD7B knockdown led to decreased RIPK1 protein levels, accompanied by a corresponding reduction in RIPK1 phosphorylation, is consistent with the possibility that OTUD7B may stabilize RIPK1 by removing K48-linked polyubiquitin chains, which typically target proteins for proteasomal degradation, thereby expanding the substrate pool available for phosphorylation and amplifying downstream necroptotic signaling (33), or alternatively, enhance its activation by promoting K63-linked ubiquitination (34). This modulation would potentiate RIPK1's interaction with downstream signaling components, thereby driving the necroptotic cascade in AS. However, this proposed mechanism remains hypothetical and requires further validation through site-specific ubiquitination assays, which would significantly enrich our understanding of the post-translational regulatory network governing necroptosis. Supporting the relevance of this pathway in AS, plasma levels of RIPK1, RIPK3, and MLKL have been found to be closely associated with coronary heart disease (CHD) and possess predictive value for the prognosis of patients with CHD caused by coronary atherosclerosis (35). Moreover, inhibiting the expression of RIPK1, RIPK3, and MLKL in endothelial cells has been shown to suppress atherosclerotic necroptosis (36).

The clinical significance of this study is twofold. It identifies OTUD7B as a novel pro-atherogenic factor, offering a potential new biomarker for atherosclerosis risk assessment. Additionally, it demonstrates that targeted inhibition of OTUD7B attenuates atherosclerosis lesions by suppressing RIPK1-mediated necroptosis, providing a strong theoretical foundation for developing novel atherosclerosis treatment strategies. Compared to conventional anti-inflammatory or lipid-lowering therapies, targeting OTUD7B offers the potential advantage of more specifically disrupting the necroptosis-inflammation vicious cycle, potentially minimizing systemic side effects.

However, several limitations still warrant acknowledgment. Firstly, while VSMC-specific OTUD7B knockdown was achieved using the SM22α promoter, the expression and functional role of OTUD7B in other critical atherosclerosis cell types, such as endothelial cells and macrophages, remain unexplored. Necroptosis in these cells also significantly contributes to lesion progression (37). Secondly, although our Co-IP experiments confirmed a direct interaction between OTUD7B and RIPK1, and functional studies demonstrated that OTUD7B knockdown reduces RIPK1 protein levels, direct evidence of OTUD7B-mediated deubiquitination of RIPK1 is currently lacking. The specific types of ubiquitin linkage (e.g., K48, K63, M1) and the precise residues on RIPK1 modified by OTUD7B require definitive identification through ubiquitination assays and mass spectrometry analysis. This limitation means that while we propose OTUD7B modulates RIPK1 stability via deubiquitination, the precise molecular mechanism remains to be fully elucidated. Moreover, the conclusions are primarily derived from mouse models, so validation of OTUD7B's expression pattern and clinical relevance in human atherosclerosis tissues requires large-scale clinical studies. In the future, research should focus on elucidating the cell-type-specific functions of OTUD7B using conditional knockout models, developing specific OTUD7B inhibitors and evaluating their therapeutic efficacy in preclinical atherosclerosis models, and analyzing the association between OTUD7B expression levels and clinical outcomes in atherosclerosis patients (e.g., plaque stability, cardiovascular events) to facilitate clinical translation.

In conclusion, this study establishes that OTUD7B exacerbates atherosclerosis by directly binding to RIPK1, activating the RIPK1/RIPK3/MLKL pathway, and thereby promoting VSMC necroptosis and inflammation. Targeted inhibition of OTUD7B represents a promising strategy to impede atherosclerosis progression by blocking this pathway, offering a novel molecular target and therapeutic paradigm for combating this prevalent cardiovascular disease.

## Conclusion

Collectively, this study demonstrates that OTUD7B expression is significantly upregulated in AS. Genetic knockdown of OTUD7B effectively suppresses necroptosis in VSMCs, attenuates atherosclerotic lesions, and reduces inflammatory responses. This protective effect is predominantly mediated through the RIPK1-dependent necroptosis signaling pathway. These findings identify OTUD7B as a potential pathogenic target regulating necroptosis and suggest that its inhibition represents a novel therapeutic approach for atherosclerosis treatment.

## Data Availability

The original contributions presented in the study are included in the article/Supplementary Material, further inquiries can be directed to the corresponding author.

## Supplementary material

Supplementary Figure S1 Serum lipid profiles in Control and AS groups. Serum levels of total cholesterol (TC), triglycerides (TG), and low-density lipoprotein cholesterol (LDL-C) were measured. Data are presented as mean ± SD (n = 6 per group). ns, not significant (*P* > 0.05) by unpaired *t*-test.
