# Supplementary figures and images for: OTUD7B exacerbates atherosclerosis by promoting RIPK1-dependent vascular smooth muscle cell necroptosis
Source: Front Cardiovasc Med. 2026 Jun 18;13:1749659. doi: 10.3389/fcvm.2026.1749659 (PMC13322888; doi:10.3389/fcvm.2026.1749659)

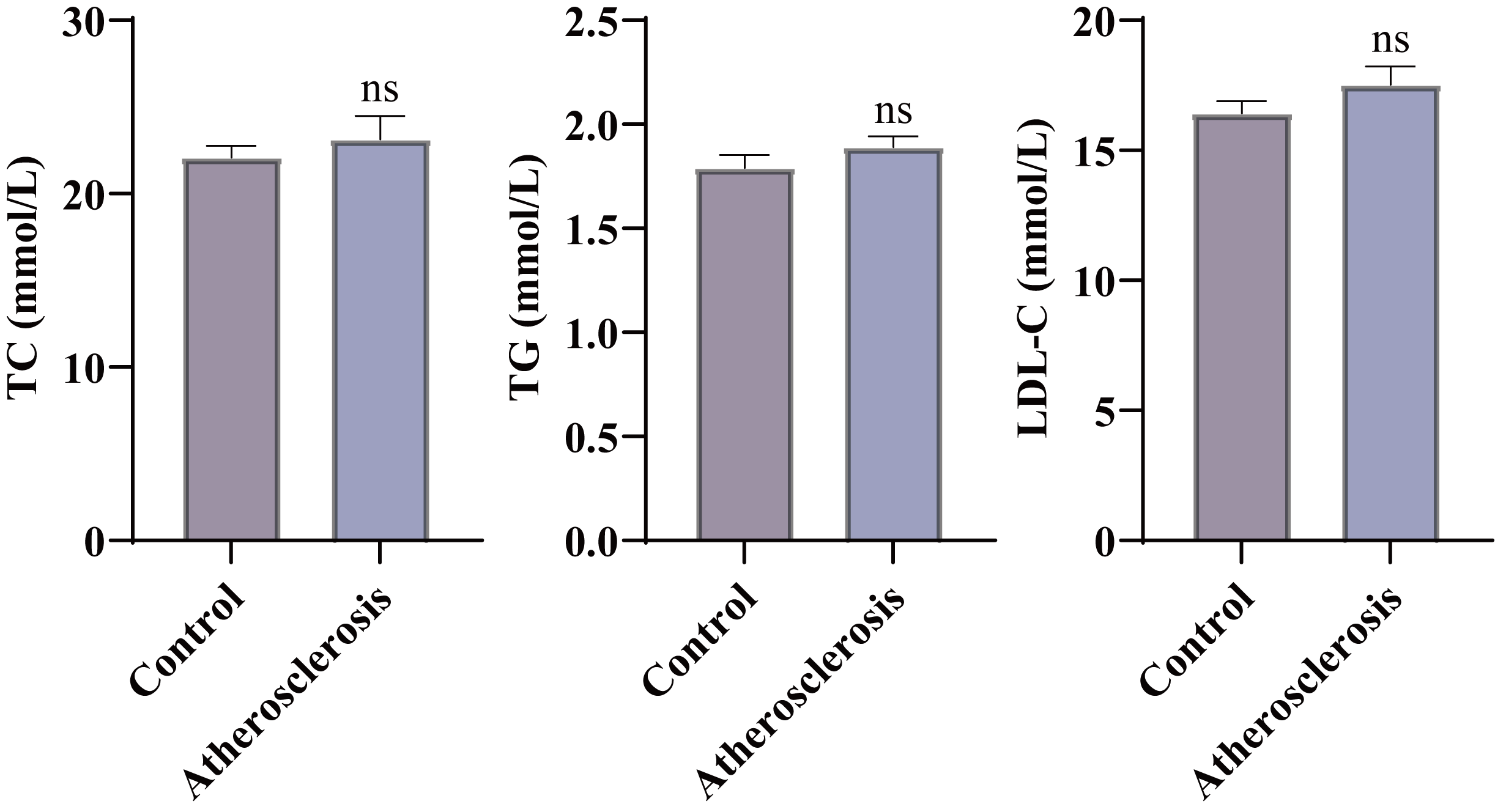

Supplement: Supplementary Figure S1 — Serum lipid profiles in Control and AS groups. Serum levels of total cholesterol (TC), triglycerides (TG), and low-density lipoprotein cholesterol (LDL-C) were measured. Data are presented as mean ± SD (n = 6 per group). ns, not significant (P > 0.05) by unpaired t-test. [file Image1.tif]
