# Supplementary material for: OTUD7B exacerbates atherosclerosis by promoting RIPK1-dependent vascular smooth muscle cell necroptosis
Source: Front Cardiovasc Med. 2026 Jun 18;13:1749659. doi: 10.3389/fcvm.2026.1749659 (PMC13322888; doi:10.3389/fcvm.2026.1749659)
Supplement: Supplementary file 2 [file Table1.docx]

**
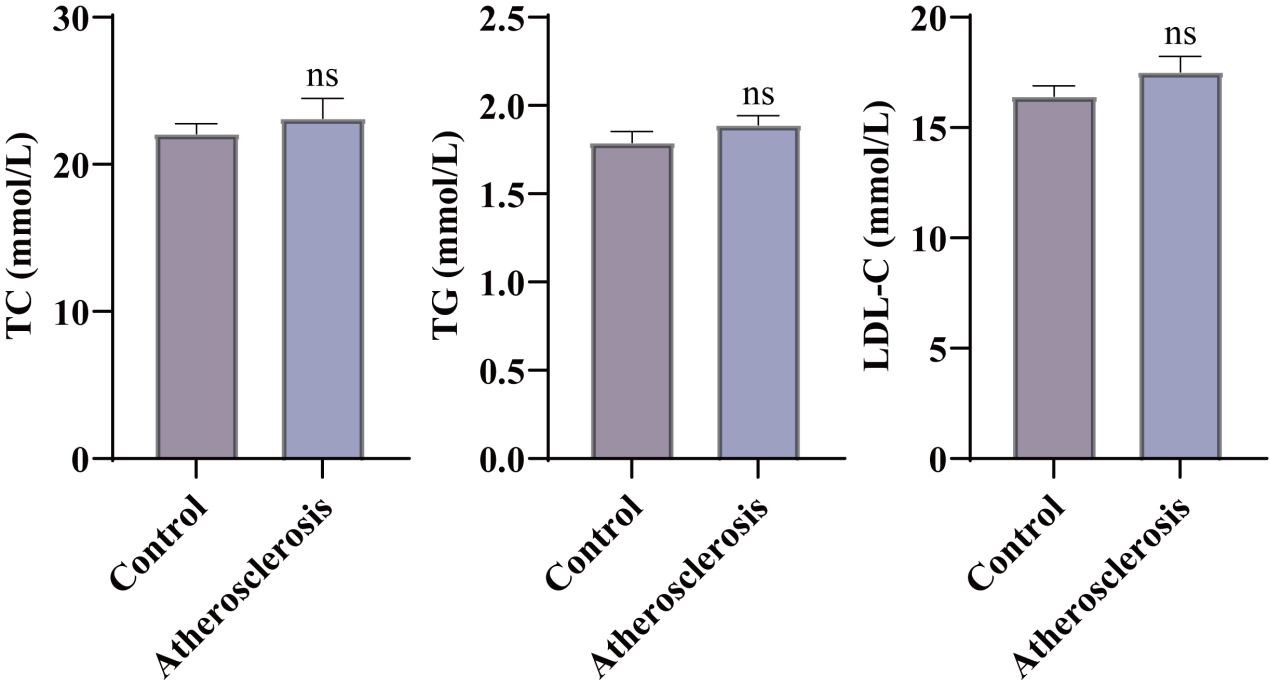
**

**Fig. S1 Serum lipid profiles in Control and AS groups.**

Serum levels of total cholesterol (TC), triglycerides (TG), and low-density lipoprotein cholesterol (LDL-C) were measured. Data are presented as mean ± SD (n=6 per group). ns, not significant (P > 0.05) by unpaired t-test.
